# Epigenetic and Phenotypic Profile of Fibroblasts Derived from Induced Pluripotent Stem Cells

**DOI:** 10.1371/journal.pone.0017128

**Published:** 2011-02-28

**Authors:** Kyle J. Hewitt, Yulia Shamis, Ryan B. Hayman, Mariam Margvelashvili, Shumin Dong, Mark W. Carlson, Jonathan A. Garlick

**Affiliations:** 1 Program in Cell, Molecular and Developmental Biology, Sackler School of Graduate Biomedical Sciences, Tufts University School of Medicine, Boston, Massachusetts, United States of America; 2 Department of Chemistry, Tufts University, Medford, Massachusetts, United States of America; 3 Department of Oral and Maxillofacial Pathology, Tufts University, Boston, Massachusetts, United States of America; 4 Department of Dental Materials, School of Dentistry, University of Siena, Siena, Italy; University of Minnesota, United States of America

## Abstract

Human induced pluripotent stem (hiPS) cells offer a novel source of patient-specific cells for regenerative medicine. However, the biological potential of iPS-derived cells and their similarities to cells differentiated from human embryonic stem (hES) cells remain unclear. We derived fibroblast-like cells from two hiPS cell lines and show that their phenotypic properties and patterns of DNA methylation were similar to that of mature fibroblasts and to fibroblasts derived from hES cells. iPS-derived fibroblasts (iPDK) and their hES-derived counterparts (EDK) showed similar cell morphology throughout differentiation, and patterns of gene expression and cell surface markers were characteristic of mature fibroblasts. Array-based methylation analysis was performed for EDK, iPDK and their parental hES and iPS cell lines, and hierarchical clustering revealed that EDK and iPDK had closely-related methylation profiles. DNA methylation analysis of promoter regions associated with extracellular matrix (ECM)-production (COL1A1) by iPS- and hESC-derived fibroblasts and fibroblast lineage commitment (PDGFRβ), revealed promoter demethylation linked to their expression, and patterns of transcription and methylation of genes related to the functional properties of mature stromal cells were seen in both hiPS- and hES-derived fibroblasts. iPDK cells also showed functional properties analogous to those of hES-derived and mature fibroblasts, as seen by their capacity to direct the morphogenesis of engineered human skin equivalents. Characterization of the functional behavior of ES- and iPS-derived fibroblasts in engineered 3D tissues demonstrates the utility of this tissue platform to predict the capacity of iPS-derived cells before their therapeutic application.

## Introduction

Human induced pluripotent stem (hiPS) cells have great potential to generate patient-specific cells that may serve as a robust source of progenitors for regenerative medicine. It has recently been shown that iPS cells are similar in their patterns of gene expression and epigenetic profile to embryonic stem cells [1], in spite of evidence showing phenotypic differences between them [2]–[5]. In this light, it is particularly important to determine if specific cell types derived from iPS and human ES (hES) cells, using the same derivation strategies, will generate cells with similar functional features. Using protocols initially established for hES cell differentiation, it has been shown that hiPS reprogrammed from adult fibroblasts can be directed into specific cell types and lineages [4], [6]–[8]. However, it remains unclear if the molecular and cellular features that direct the biological potential and functional behavior are restored in a characteristic manner once hiPS cells are differentiated towards a fibroblast lineage.

We have recently reported a protocol to efficiently derive cells from hES cells that show phenotypic and functional features of human stromal fibroblasts [9]. Stromal fibroblasts support the development, repair and homeostasis of their resident tissues [10] and understanding their differentiation from hES and hiPS will be critical to designing effective strategies for their use in future regenerative therapies [11]. Several methods have been established to generate cells with features of mesenchymal stem cell (MSC)-like cells from hES cells [12]–[14]. However, due to an incomplete understanding of fibroblast development from MSCs and to their cellular heterogeneity *in vivo* that results in a lack of definitive markers needed to isolate them [15], [16], realization of the therapeutic potential of fibroblasts has been limited [17]. In light of this, stromal fibroblasts derived from hES or iPS may serve as an alternative source of more uniform, well-characterized stromal cells that can offer predictable tissue outcomes. Beyond this, the possibility that MSCs derived from iPS might acquire a biological potency that would exceed that of the fibroblasts from which they were originally derived [18]–[20], raises further interest in characterizing iPS as a source of stromal fibroblasts.

During the process of reprogramming of human somatic cells to hiPS, cells undergo dramatic epigenetic changes that include recalibration of DNA methylation that resets transcriptional programs [1], [21]. The subsequent differentiation of iPS cells to mature cell types is dependent on reestablishment of methylation marks that govern patterns of gene expression to give stable, functional cell types [22], [23]. As a result, mature cell types derived from these pluripotent sources acquire unique DNA methylation signatures associated with their phenotype [24], [25]. Analysis of the differential methylation between human pluripotent stem cells and cells differentiated from them hold promise for revealing the underlying basis for how lineage specification occurs upon activation of developmental programs [26]. DNA methylation analyses can also be used to compare the normalization of iPS-derived cells to the somatic cell population from which iPS were originally reprogrammed. For this reason, determining the similarities or differences in the status of CpG promoter methylation for a specific cell type differentiated from both hES and hiPS is important for their characterization. Whole-genome DNA methylation analysis has been used recently to compare methylation and associated patterns of gene expression between hES and cells derived from them [27], and has demonstrated a global shift towards methylation and silencing of subsets of genes upon differentiation to specific lineages. However, such analysis has not been performed to compare human cells derived from ES and iPS that demonstrate the functional properties of mesenchymal cells or stromal fibroblasts.

In the present study, we generated cells with properties of stromal fibroblasts from multiple hiPS cell lines that were initially reprogrammed using two different methods, and showed that their phenotype was similar to both mature fibroblasts and to hES-derived fibroblasts. We found that iPS-derived fibroblasts (iPDK) and their hES-derived counterparts (EDK) shared similar cell morphology through discrete stages of differentiation, and displayed patterns of gene expression and cell surface markers characteristic of mature fibroblasts. All iPS- and hES-derived cell lines showed functional properties of fibroblasts, as seen by their capacity to direct epidermal morphogenesis upon incorporation into the stromal compartment of engineered human skin equivalents. Characterization of gene promoter methylation in EDK, iPDK, hES and iPS cell lines performed by array-based analysis of the methylation status of over 27,000 CpG sites in these cell types, revealed a shift towards methylation and silencing of specific genes upon differentiation. We have linked this characterization to the behavior of ES- and iPS-derived cells in engineered 3D tissues to demonstrate the function of fibroblasts derived from iPS in *in vivo*-like tissue environments that may predict their capacity for regenerative medicine and use in disease models.

## Results

### ES- and iPS-derived cells demonstrate morphologic and phenotypic features of fibroblast-like cells

**Figure 1:**
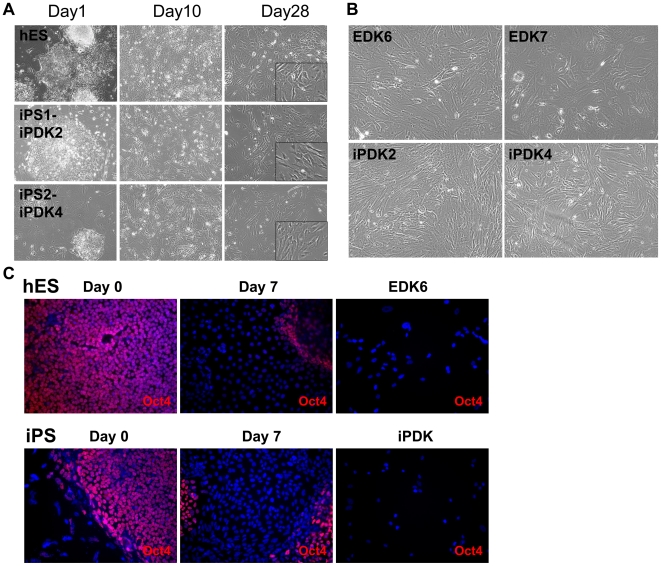
Morphology of ES and iPS cells during sequential stages of differentiation. hES and hiPS cells were induced to differentiate in parallel, using identical differentiation procedures, and monitored for cell morphology at various stages. Representative images of hES and 2 iPS lines during differentiation showed similar morphology at days 1, 10, and 28 of differentiation (A). iPS differentiation resulted in fewer surviving cells at each stage, yet occurred on a similar time course to hES differentiation. Stable cell lines also had similar morphology at passage 8 of differentiation (B). All images taken at 10X magnification. Immunohistochemistry during the early stages of differentiation showed the progressive loss of pluripotency-related protein Oct-4, and Oct-4 was undetectable in differentiated cell types (C). Images taken at 20X magnification.

hiPS cells were initially generated and characterized by the Hochedlinger lab using retroviral transduction of BJ fibroblasts (ATCC, Inc., Manassas, VA) with five retroviral reprogramming factors (BJ hiPS#1) [28], and additional cells were also used that were reprogrammed using four retroviral reprogramming factors (BJ iPS #2, LD iPS). We derived cells from iPS (iPDK2 derived from BJ hiPS #1 and iPDK3 and iPDK4 from BJ iPS #2) using a differentiation protocol that was previously used to generate cells with properties of human fibroblasts from hES cells (H9 hES cell line) [9]. In parallel, hES cells were used to derive new fibroblast cell lines (EDK6 and EDK7) using the same differentiation conditions. Cells derived from both hES and iPS demonstrated a similar appearance during the sequential stages of differentiation as seen 10 and 28 days after initiation of differentiation (**Figure 1A**) and 4, 7, 14, and 21 days following differentiation (**Figure S1**). At 28 days after differentiation, iPDK and EDK cell lines showed similar fibroblast morphology, characterized by cells with elongated and stellate shapes that maintained this morphology over prolonged passage (**Figure 1B**). During differentiation, we observed the progressive loss of pluripotency marker OCT4 in both hES and iPS cells, and this marker was absent in fully-differentiated cells (Figure 1C). Additionally, cell surface markers of hES and iPS cells, TRA-1-60 and SSEA4, were lost upon induction of differentiation, and were undetectable in stable EDK and iPDK cell types (**Figure S1B**). All four cell populations (EDK6, EDK7, iPDK2, iPDK4) showed similar expression of CD markers, CD10, CD13, CD44, CD73, CD90, and CD166, characteristic of MSCs and fibroblasts (**Table 1**). These surface markers were found in greater than 90% of EDK6, EDK7, iPDK2, iPDK4 cells and were comparable to the percentage of cells expressing these markers in mature BJ fibroblasts, suggesting a biological potential for hES and iPS-derived cells that is similar to stromal fibroblasts. The growth of these cells was stable over long-term culture as seen by the maintenance of cell numbers upon serial passage and was similar to growth seen in normal fibroblasts (**Figure S2**). Taken together, this data indicates that hiPS cells can readily and reproducibly differentiate to cells with a gene expression profile of fibroblasts in a similar manner to that identified for hES cells.

**Table 1:** Flow Cytometric Analysis of ES- and iPS-derived Cells.

Marker	Control	ES-derived		iPS-derived	
	BJ	EDK6	EDK7	iPDK2	iPDK4
CD10	99.8	98.1	100	99.9	96.5
CD13	100	99.7	96.7	90.2	99.1
CD44	99.9	96.2	99.9	99.9	99.8
CD73	99.8	99.8	99.9	99.9	99.7
CD90	100	90.4	100	100	99.9
CD166	99.7	98.8	99.9	99.9	99.4

The percentages of cells positive for the cell surface markers are shown. Each experiment is normalized to isotype control, and has been repeated at least 2 times.

### Methylation changes upon differentiation reveal characteristic features of commitment to a fibroblast lineage in both iPDK and EDK cell lines

Since changes in differentiation state of pluripotent-derived cells are often associated with DNA methylation [25], we profiled changes in DNA methylation of CpG sites in both EDK and iPDK upon their differentiation. We compared their methylation state using an Illumina Methylation27 chip, which analyzes 27,000 DNA methylation sites in a large range of gene promoters both within and outside of CpG islands. The status of each methylation site within this data set is represented by a number from 0 to 1.0, indicating the fraction of methylated cells present in relation to the total number of cells within the population.

**Figure 2:**
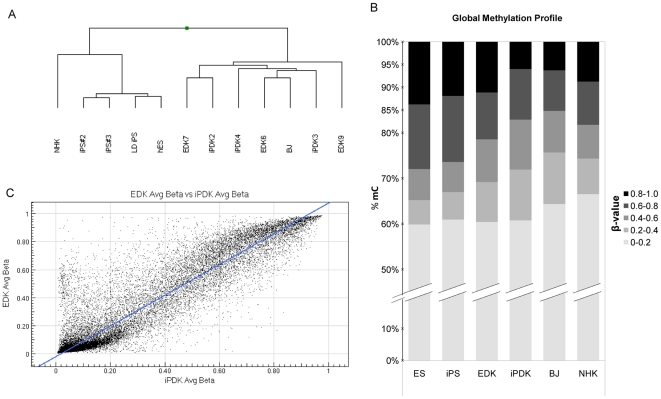
Global profiling of DNA methylation indicates that EDK and iPDK cells share similar gene methylation levels that are distinct from pluripotent cell types. Cell lines were analyzed for methylation marks using the Illumina Methylation27 chip. Hierarchical clustering analysis of degrees of methylation using a Manhattan correlation showed that hES and iPS cell lines clustered in a distinct set from differentiated iPDK and EDK cells, as well as mature fibroblasts and keratinocytes (A). The methylation status of each cell type, as assessed by the percentage of CpG sites that fell within a range of β-values, showed that CpG islands are over 60% unmethylated, and pluripotent cells appear to have greater number of highly-methylated CpG sites (B). A scatter plot comparing the mean level of CpG methylation in 3 EDK and 3 iPDK cell lines showed a high degree of correlation between the two cell types (C). R^2^ = 0.8715.

Hierarchical clustering analysis of methylation data revealed that all of the EDK and iPDK cell types had closely-related methylation profiles, while all pluripotent cell types clustered together and were distinct from EDK and iPDK (**Figure 2A**). Normal human fibroblasts (HFF) clustered closely with the EDK and iPDK samples, while the foreskin-derived keratinocyte (NHK) methylation profile was more similar to pluripotent cells studied, unrelated to fibroblasts. Additionally, the degree of total methylation for all the sites represented in this assay was compared between cell samples by counting the number of CpG sites falling within a range of β values varying in degree from highly methylated (0.8–1.0) to unmethylated (0–0.2) (**Figure 2B**). Methylation was found to be reduced in differentiated EDK and iPDK cells when compared to the pluripotent cell types from which they were derived. A scatter plot illustrating the comparative methylation state of both EDK and iPDK showed a high degree of correlation (R^2^ = 0.87) indicating that the methylation profile was similar between these two cell types (**Figure 2C**).

**Figure 3:**
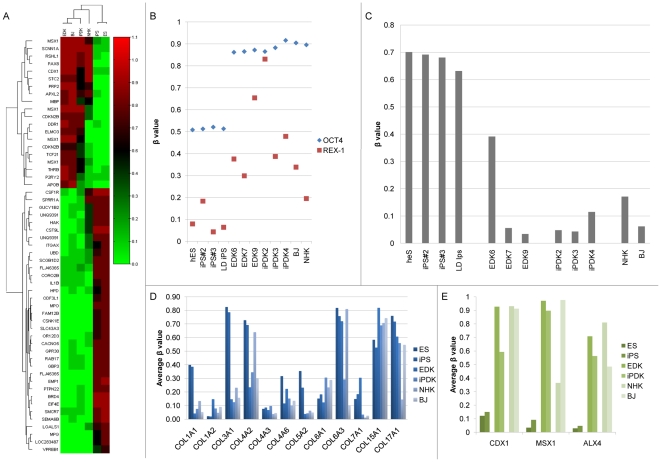
Changes in DNA methylation of gene promoter regions following differentiation shows large changes in methylation status in EDK and iPDK compared to hES and iPS. Heatmap showing 55 CpG sites which were differentially methylated by more than 70% in both EDK and iPDK when compared to their hES cell counterparts (A). In addition, the pluripotency-related genes OCT4 and REX1 showed increased CpG methylation in EDK and iPDK cells compared to hES and iPS cells (B). Analysis of Collagen promoter methylation showed that the COL1A1, COL3A1 and COL4A2 promoters were demethylated upon differentiation towards fibroblasts while collagen genes expressed in epithelial cell types, such as COL17A1, remained methylated in differentiated cell types (C). Fibroblast-related gene PDGFR-β was demethylated in differentiated cell types (D). Differentially-methylated Hox genes, grouped by cell type, showed a distinct methylation of Hox genes related to mesenchymal lineage commitment (E).

We next examined the genes that showed the greatest difference in methylation status between undifferentiated pluripotent and differentiated fibroblast cell types, and generated a heat map to compare the average methylation status of EDK (n = 3), iPDK (n = 3), ES (n = 1), iPS (n = 3) HFF (n = 1) and NHK n = 1). We identified a total of 55 gene-specific methylation sites that showed greater than a 70% difference in methylation between ES and EDK, and a high degree of similarity to iPDK and HFF (**Figure 3A**). Only 51 gene promoter regions are represented in this dataset, as MSX1 (3 times) and CDKN2B (2 times) appeared on multiple occasions in the array. Methylation of specific promoter regions of genes associated with pluripotency were also assessed using this screen. The OCT4 and REX1 promoter CpG sites showed consistently elevated levels of methylation upon differentiation towards fibroblasts, indicating epigenetic silencing of pluripotency genes as cells underwent commitment towards stable, differentiated cell types (**Figure 3B**). We then analyzed the methylation status of a group of promoter regions associated with the expression of collagen genes, which are linked to the phenotypic properties of extracellular matrix (ECM)-producing fibroblasts. Several of the collagen promoter regions were demethylated in EDK and iPDK cells when compared to hES and hiPS (COL1A1, 3A1, 4A2) (**Figure 3C**). Epithelial-related collagens, such as COL17A1, remained unchanged in methylation status in EDK and iPDK cells, and were only found to be demethylated in mature keratinocytes. Significantly, we found that platelet-derived growth factor-β (PDGFRβ) was consistently hypomethylated in all cells with properties of stromal fibroblasts, EDK and iPDK cells, indicating a commitment to a fibroblast lineage fate (**Figure 3D**). Several homeobox genes, CDX1, MSX1, and ALX homeobox 4 (ALX4), known to regulate various stages of mesenchymal differentiation, all showed increased promoter methylation in EDK and iPDK cells when compared to hES cells, and were methylated at a comparable level to that seen in mature fibroblasts (**Figure 3E**). CDX1 promoter has been previously identified as a hypermethylated region in fibroblasts derived from hES [27], while MSX1 is involved in craniofacial development and methylation at this site appears to be highly correlated to mesenchymal cell types [29]. The occurrence of DNA methylation in EDK and iPDK at multiple sites within the MSX1 promoter may indicate an extended region of hypermethylation.

### Gene expression of fibroblast-related genes is similar between iPDK and EDK cell lines and correlates with methylation data

**Figure 4:**
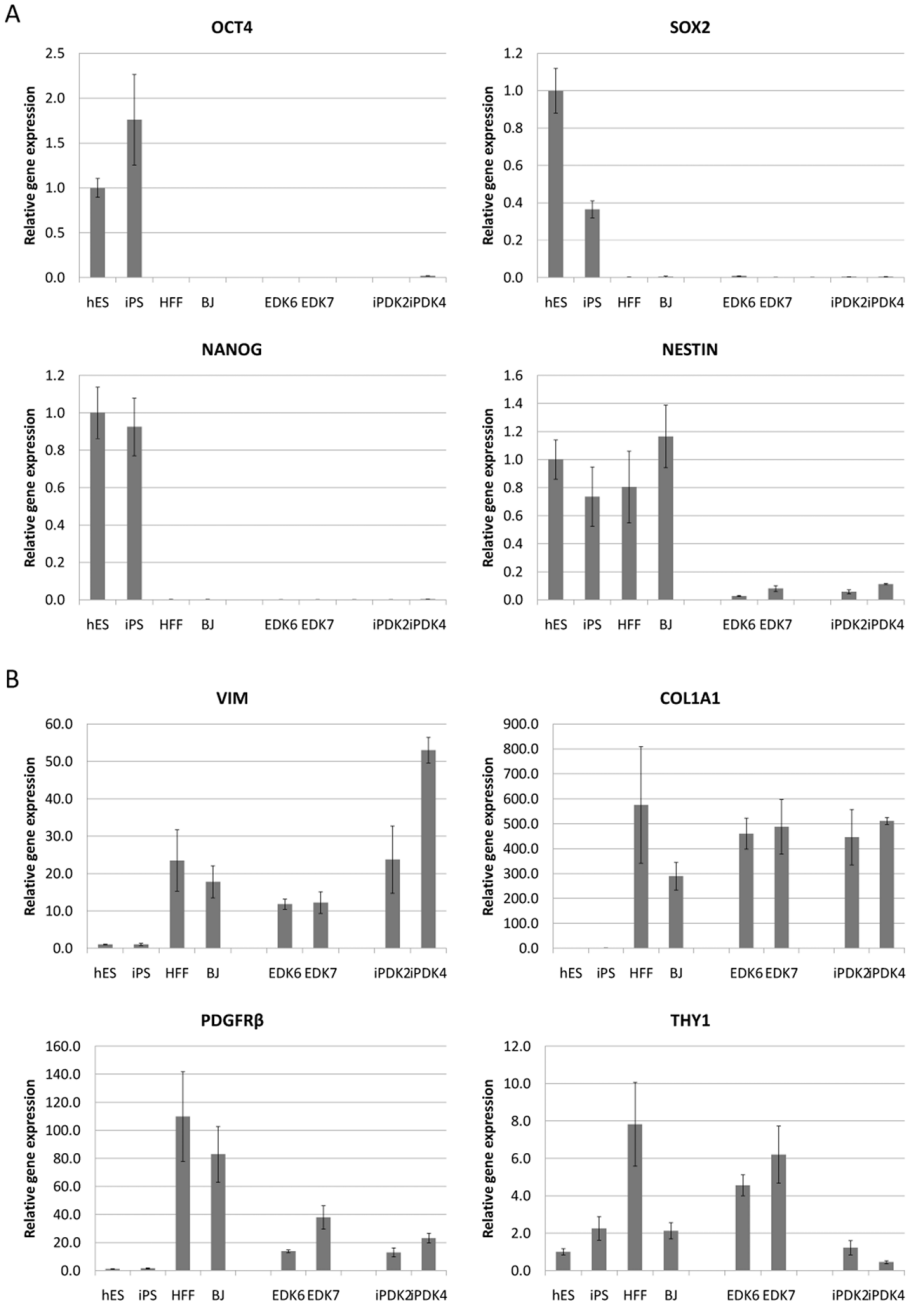
Gene expression of EDK and iPDK are similar to normal fibroblasts and distinct from pluripotent cells. hES and hiPS cells as well as EDK, iPDK, HFF and BJ were analyzed by real-time RT-PCR (n = 3) and values were normalized to expression in hES cells. The pluripotency gene OCT4, SOX2 and NANOG were expressed in ES and iPS cells, and were absent in all ES and iPS-derived cells, and EDK and iPDK cells did not express neural stem cell marker Nestin (A). In contrast, genes characteristic of fibroblasts, including vimentin (VIM) Type I collagen (COLIA1) and platelet-derived growth factor-beta (PDGFRB) were all upregulated in EDK and iPDK cell lines compared to pluripotent cells from which they were derived (B).

To further study the properties of iPDK and EDK cells during their differentiation towards a fibroblast lineage fate, we analyzed patterns of gene expression in EDK6, EDK7, iPDK2, iPDK4 after eight passages on Type I Collagen-coated plates and compared them to undifferentiated hiPS and hES cells from which they were derived and to foreskin-derived fibroblasts (BJ and HFF). Expression of OCT4, SOX2 and NANOG were significantly down-regulated during differentiation in all EDK and iPDK cell lines when compared to hES and iPS cells, indicating loss of pluripotency upon differentiation (**Figure 4A**). Additionally, expression of the neural precursor gene nestin was significantly down-regulated in all EDK and iPDK cells upon their differentiation, suggesting they did not undergo commitment to a neural fate (**Figure 4A**). In contrast, the expression of genes commonly associated with mesenchymal cells and fibroblasts, including vimentin (VIM) and platelet-derived growth factor receptor-β (PDGFRβ), were consistently upregulated in EDK and iPDK cells following differentiation (**Figure 4B**). This data correlates with the results from the methylation screen that showed a decrease in methylation of the PDGFRβ promoter when both iPS and hES are differentiated to iPDK and EDK cell lines (**Figure 3C**). Type I collagen (COLIA, COLIA2) was also significantly upregulated in iPS- and hES-derived cell lines following their differentiation (**Figure 4B**), consistent with a decrease in the promoter methylation of COL1A1 seen in differentiated cell types (**Figure 3D**). Expression levels of VIM and COL1A1/2 were also comparable to those seen in mature fibroblasts (**Figure 4B**), while the expression levels of PDGFRβ was lower in EDK and iPDK when compared to fibroblasts. Another marker of fibroblasts, Thy-1, showed higher expression in the hES-derived cells compared to iPS-derived lines, but we also observed significant variability in expression in different lines of mature fibroblasts that were tested.

#### iPDK and EDK both support the development and maturation of keratinocytes when incorporated into the stromal compartment of human skin equivalents

**Figure 5:**
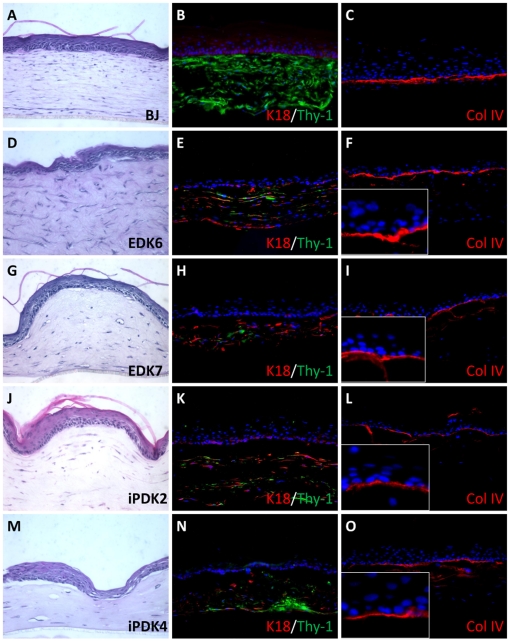
EDK and iPDK support the development of a fully-differentiated epithelium when incorporated into the stroma of 3D Skin Equivalents. Fibroblast-like cells from independent differentiations of ES and iPS cells were incorporated into a collagen gel and allowed to contract. The collagen gel was then seeded with foreskin keratinocytes and grown at an air-liquid interface. H&E staining of tissues revealed that EDK and iPDK cells within tissues displayed an elongated morphology in the collagen gel that supported the growth and differentiation of keratinocytes (D,G,J,M) similar to control foreskin-derived fibroblasts (A). Immunostaining of these tissues shows the expression of fibroblast marker Thy-1 (green) in the stromal compartment of all tissues, as well as K18 (red) within ES- and iPS-derived cells (B,E,H,K,N). Basement membrane marker, Type IV collagen (red) was localized to the region between the stromal and epidermal compartment, indicating the establishment of the basement membrane in these tissues (C,F,I,L,O).

In light of the fibroblast phenotype seen for both EDK and iPDK cells, we next studied whether these cells could contribute to normalized tissue morphogenesis of human skin equivalents (HSEs) when incorporated into the stromal compartment of engineered tissues. These tissues can serve as an *in vitro* surrogate of human skin that have been previously used to study the capacity of fibroblasts to support the growth and differentiation of the surface epithelium [30], [31]. EDK and iPDK cell lines were incorporated into collagen gels and were grown at an air-liquid interface for 10 days, with foreskin-derived, human keratinocytes (NHK) on their surface. Tissues incorporating either iPDK or EDK cells in the stromal compartment showed a similar degree of epithelial morphogenesis on their surface that included a fully-differentiated epithelium demonstrating a distinct basal layer and a multi-layer tissue showing skin-like architecture (**Figure 5D,G,J,M**). All tissues showed a similar degree of morphologic differentiation and thickness that was similar to control tissues that developed in the presence of mature fibroblasts (**Figure 5A**). All EDK and iPDK cell lines demonstrated a fibroblastic morphology in collagen gels that was similar to the morphology of HFF cells, indicating that both hES- and iPS-derived cells types behaved similarly to stromal fibroblasts in collagen gels. Immunofluorescence staining of tissues for fibroblast-specific markers showed the expression of Thy-1 in EDK and iPDK in the connective tissue (**Figure 5E,H,K**). Cytokeratin-18 (K18) was also detected in EDK and iPDK cells in both 2D cultures and 3D tissues, although it was not expressed in adult fibroblasts. The normalization of tissue phenotype and structure was seen by the linear deposition of Type IV Collagen at the stromal-epithelial interface, indicating the presence of a basement membrane (**Figure 5C,F,I,L,O**). Tissue organization and markers showed that both EDK and iPDK cell lines demonstrated a fibroblast phenotype that were able to direct the normalization of epithelial tissue phenotype.

## Discussion

We did not find major differences between stromal cells derived from iPS and those derived from hES cells when their step-wise differentiation, transcriptional program and biological potency were characterized. The similarities we observed included their cellular morphology at distinct stages of differentiation, expression of cell surface markers and proteins commonly seen in mature fibroblasts, and functional *in vitro* tissue outcomes upon their incorporation into engineered skin-like tissues. Our methylation analysis revealed that both hES- and iPS-derived fibroblasts demonstrated similar methylation patterns and showed an increase in methylation of developmentally-regulated genes when compared to pluripotent cells.

It has been shown that both hES and iPS cells have a similar potential to differentiate to specific cell types with similar morphologic features [4], [30], [32]. However, it was unclear if cells derived from these two pluripotent stem cell sources demonstrate similar molecular and cellular features linked to their biological function. For example, iPS have shown a reduced differentiation efficiency, as well as an increased variability in the potency of individual clones derived from iPS when compared to hES cells [8]. In addition, iPS-derived vascular and blood cells showed decreased growth and differentiation efficiency when compared to their hES-derived counterparts and retinal pigment epithelial cells derived from iPS underwent senescence more rapidly than cells derived from hES cells [4]. These studies demonstrated the need for further functional comparison between hES and iPS-derived cells to evaluate their similarities and differences before clinical application of these cell types.

We utilized an *in vivo-*like tissue environment to study how iPS- and hES-derived fibroblasts would behave when compared to normal fibroblasts, demonstrating the functional capacity of EDK and iPDK to support the development of a multilayer epithelium in a 3D tissue context. Several other functional readouts have been used for ES-derived cells including beating cardiomyocytes [6,6], neuronal action potentials [33], and lipid absorption in 2D monolayer cultures. However, incorporation of iPS-derived cells into engineered tissues enables a more complete determination of their lineage commitment, fate and phenotype than those evaluated in more conventional, monolayer cultures. The development of 3D, skin-like tissues *in vitro* provides a functional readout of stromal cell capacity to provide cross-talk needed for epithelial morphogenesis [30], [31]. Our findings address a central challenge facing clinical application of iPS-derived cells for regenerative medicine by providing a platform to evaluate tissue outcomes before their future therapeutic use. In conjunction with molecular characterization needed to confirm the down-regulation of pluripotency genes, these engineered tissues may serve as an important screening tool to examine the safety and stability of iPS-derived cells before transplantation. The ability to incorporate hES- and iPS-derived cells into 3D skin-like tissues provides initial proof-of-concept that may enable future screening of multiple sources of hiPS clones. In addition, the ability to compare the original fibroblast line that iPS were derived from to fibroblasts subsequently derived from them, will help establish if these differentiated cells retain a normalcy to their cell of origin. This will contribute to ongoing studies aimed at fully identifying the epigenetic regulatory elements necessary for the establishment and maintenance of differentiated cells that will need to be stable and safe for *in vivo* transplantation.

It has been shown that early-passage mouse iPS cells retain an epigenetic memory that reflects the cell type from which they were originally derived and may alter their *in vitro* differentiation potential [34]. While studies on human iPS and hES have shown they are nearly identical in their chromatin structure and patterns of gene expression [1], it is unknown if these similarities will be maintained upon their differentiation to specific cell types. With this in mind, we have performed methylation analysis of nearly 15,000 promoter regions to compare changes in methylation that occur upon differentiation from hES and iPS to fibroblasts (EDK and iPDK). We found that DNA methylation patterns were similar in both iPDK and EDK cells, and that methylation was linked to changes in expression of genes associated with their lineage commitment upon differentiation. These methylation patterns clustered with foreskin-derived fibroblasts, and they were distinct from patterns observed in undifferentiated iPS and mature adult keratinocytes. There was an overall increase in methylation in both EDK and iPDK cells within the promoter region of pluripotency-related genes, indicating a silencing of pluripotent genes that correlates with differentiation to a fibroblast phenotype and concomitant down regulation of expression of OCT4. Demethylation at the PDGFR-β promoter has been identified in previous screens comparing hES cell methylation profiles to other differentiated tissues [25], and may represent an important site of regulation during differentiation. Similar differences in methylation have also been reported when the parental h9-hES cells were compared to fibroblast-like cells that had spontaneously differentiated from them [27]. We extend these findings here using iPS-derived fibroblasts that allowed a more complete characterization by comparing them to mature, human fibroblasts prior to reprogramming. However, it is still unknown whether specific CpG methylation sites are responsible for any differences in functionality we observed in our 3D culture system. An important avenue of future research will be to identify specific epigenetic marks that can link sub-types, such as the fibroblasts we have derived, with their functional properties.

Since numerous tissue types require the support of stromal fibroblasts to provide appropriate cross-talk and ECM production to maintain tissue homeostasis and enable repair, the fibroblast cells that we derived have great potential for applications in regenerative medicine [35]. While adult MSCs hold potential for the isolation of such fibroblasts, their limited capacity to proliferate and loss of differentiation potential during *in vitro* expansion restricts their ability to serve as a source of fibroblasts. Pluripotent stem cells offer an attractive alternative source for stromal fibroblasts. Cells with phenotypic properties of MSCs have previously been derived from hES and iPS cells by several approaches [18], [36]. These studies have derived MSC-like cells that have shown biological potencies similar to adult MSC, but have not addressed the need to differentiate and assess the function of stromal fibroblasts. Human MSCs, some of which are found in the stroma of skin and other tissues, have previously been shown to express low molecular-weight cytokeratins, such as K18 [37]. Therefore, it is possible that expression of K18 in EDK and iPDK cells reflects the phenotypic overlap of EDK and IPDK cells with MSC-like cells.

Our demonstration that iPS-derived cells can be differentiated to cells with functional features of stromal fibroblasts suggests they harbor a therapeutic potential that is distinct from MSC-like cells. While our analysis has revealed that iPS- and hES-derived fibroblasts are distinct from the pluripotent cells from which they were derived, it is possible that a small number of latent, pluripotent cells may be retained in these differentiated cell populations that were not identified by immunofluorescence or RT-PCR. The therapeutic implications of such residual cells have been lessened by the existence of several recently-developed reprogramming techniques that utilize excisable or non-integrating viral vectors that may further improve the safety of these cells for human transplantation. Beyond this, the growth of these differentiated cells in engineered 3D tissues may also increase our understanding of the fate and safety of hES- or iPS-derived cells before their *in vivo* therapeutic application. Since the aging process is known to limit the differentiation potential and survival of adult MSCs [38], iPS-derived cells with features of fibroblasts may be an important source for future therapeutic applications to augment repair and regeneration. Further elucidating the molecular features that define these iPS- and ES-derived fibroblasts following differentiation will allow us to fine-tune the engineering of specific types of fibroblasts that function in the development of organs and tissues.

## Materials and Methods

### Cell Culture

hiPS lines were expanded and maintained under the same conditions as described for maintenance of hES cells [39]. BJ hiPS #1 were derived and characterized as described [28], and new lines of hiPS cells were provided by Dr. Laurence Daheron, and generated from human foreskin cells (BJ cell line) (ATCC, Inc., Manassas, VA). The H9 line of hES cells used in this study were purchased from the WiCell Institute (Madison, WI). All patient-derived cells used for this study were derived from anonymous donors and are not identifiable, and thus are exempt from IRB approval. For directed differentiation, iPS or hES cells were plated onto fomaldehylde-fixed MEFs, fed with NHK media, consisting of 3∶1 DMEM:F12 (Invitrogen, Carlsbad, CA), 5% FCII (Hyclone, Logan, UT), 0.18 mM adenine, 8 mM HEPES, 0.5 µg/mL hydrocortisone, 10^−10^ M cholera toxin, 10 ng/mL EGF, 5 µg/mL insulin, and supplemented on days 4–7 with 0.5 nM BMP-4. Subsequently, cells were propagated sequentially for an additional 7 days on fixed MEFs, plastic and Type I collagen (BD Biosciences, San Jose, CA). Using this protocol, cells from 2 distinct iPS lines were differentiated and compared to H9-hES cells differentiated in parallel. iPDK2 were derived using the iPS cell line BJ hiPS#1, established in the lab of Konrad Hochedlinger [40] using separate dox-inducible vectors Oct4, Sox2, Klf4, cMyc and Nanog. iPDK3 and 4 were derived from separate clones of hiPS cells, which were reprogrammed using 4 reprogramming factors (Harvard Stem Cell Institute).

### Real-time RT-PCR

RNA was extracted using the Qiagen RNeasy kit. The cDNA was transcribed with 0.5 µg RNA using the Quantiscript Reverse Transcriptase (Qiagen, Valencia, CA). For each real-time RT-PCR reaction, we used 20 ng of cDNA, 200 nM of each primer, and 2X SYBRgreen (Applied Biosystems, Foster City, CA) and samples were run on the Bio-Rad CFX96 Real-Time PCR Detection System according to manufacturers' instructions. The relative level of gene expression was assessed using the 2^-ΔΔCt^ method and results are presented as an average of 3 experiments. Error bars represent standard deviation of a relative quantity, and were calculated using formulas provided in the Bio-Rad Instruction Manual. The following oligonucleotide primer sequences were used: GAPDH-F1 – 5′ tcgacagtcagccgcatcttcttt 3′, GAPDH-R1 – 5′ accaaatccgttgacctt 3′ nestin-F1 – 5′ tgaagggcaatcacaacagg, nestin-R1 – 5′ tgaccccaacatgacctctg 3′, OCT4-F – 5′ AGCGAACCAGTATCGAGAAAC 3′, OCT4-R – TTACAGAACCACACTCGGAC, THY1-F – 5′ CCCGCAATCCCTCAAACCT 3′, THY1-R – 5′ GCAAGGATGACCCCTCCAGT 3′, PDGFRβ-F – 5′ gtggtgatctcagccatcct 3′, PDGFRβ-R - 5′ ccgacataagggcttgctt 3′, VIM-F – 5′ attccactttgcgttcaagg 3′, VIM-R – 5′ cttcagagagaggaagccga 3′, COL1A1-F – 5′ cctcctgacgcacggccaag 3′, COL1A1-R – 5′ ccctcgacgccggtggtttc 3′, SOX2-F – 5′ GTTGTCAAGGCAGAGAAGAG 3′, SOX2-R – 5′ GAGAGAGGCAAACTGGAATC 3′, NANOG-F – 5′ GAACTCTCCAACATCCTGAACCTC 3′, NANOG-R 5′ CCTTCTGCGTCACACCATTGC 3′.

### 3-Dimensional (3D) tissue fabrication

Control tissues were grown using human keratinocytes (NHK) derived from foreskin that were first cultured in 2D, monolayer culture on irradiated 3T3 fibroblasts. Foreskin fibroblasts were grown in media containing Dulbecco's Modified Eagle's Medium (DMEM) and 10% fetal calf serum. Tissues were grown by adding foreskin fibroblasts or ES-derived, EDK1 cells to neutralized Type I Collagen (Organogenesis, Canton, MA) to a final concentration of 2.5×10^4^ cells/ml. Three ml of this mixture was added to each 35 mm well of a 6 well plate (Organogenesis, Canton, MA) and incubated for 7 days in media containing DMEM and 10% fetal calf serum, until the collagen matrix showed no further shrinkage. 5×10^5^ NHK cells were seeded onto the collagen matrix and tissues were maintained submerged in low-calcium epidermal growth media for 2 days, submerged for 2 days in normal calcium media, and then raised to the air–liquid interface for an additional 8 days by feeding from below as previously described [30].

### Immunocytochemistry and frozen section staining

EDK and iPDK cells were grown on Type I Collagen-coated coverslips, fixed in 4% paraformaldehyde and permeabilized using 0.1% Triton X-100. Staining was performed using primary antibodies directed against OCT-4 (Santa Cruz, Santa Cruz, CA), TRA-1-60 (Abcam, Cambridge MA), SSEA-4 (Millipore, Billerica, MA), K18 (Abcam, Cambridge, MA), or Thy-1 (Millipore, Billerica, MA) for 1 hour followed by Alexa 488-conjugated goat anti-rabbit or Alexa 594-conjugated goat anti-mouse secondary antibodies (Invitrogen, Carlsbad, CA) for 1 hour. For immunohistochemistry, tissues were frozen in O.C.T. compound (Sakura Finetek USA, Torrance, CA) and 8 µm sections were fixed in 4% paraformaldehyde and immunostained using the same protocol as above. Coverslips and tissues were counterstained with DAPI in Vectashield mounting medium (Vector, Burlingame, CA). Hematoxylin and Eosin (H&E) staining for morphologic analysis was performed on paraffin-embedded tissues that were sectioned at 8 µm thickness. Images were captured using a SPOT RT camera and a Nikon Eclipse 80*i* microscope.

### Flow Cytometry

EDK and iPDK cell lines were harvested, resuspended in 2% FBS in PBS, and stained with PE-conjugated anti -CD73, -CD10, -CD13, -CD44, -CD73, CD166, and -IgG1k (BD Pharmingen). Cells were incubated for 40 minutes at 4°C in the dark and washed with 2% FBS in PBS solution. All data was generated using a FACSCalibur (Becton Dickinson) and analyzed using CellQuest (Becton Dickinson) and Summit V4.3 software (Dako). Analysis was performed on 200,000 cells per sample and positive expression was defined as the level of fluorescence greater than 90% of the corresponding isotype control IgG1k (BD Biosciences) or the corresponding unstained cell samples.

### DNA Methylation Profiling

Methylation analysis was performed using the Infinium Human Methylation27 BeadChip (Illumina Inc.), analyzing 27,578 individual CpG sites. EDK and iPDK cell lines were expanded for 6 passages on Type I collagen to establish stable cell lines. Undifferentiated hES and iPS cell lines, as well as NHK and HFF cell types, were used as control and reference samples. DNA was extracted using the DNeasy kit (QIAgen) and bisulfite-converted using the EZ DNA Methylation Kit (Zymo). Converted DNA was processed and hybridized to the Infinium Methylation Chip according to manufacturers' instructions, scanned on an Illumina BeadArray Reader (Illumina Inc.) and analyzed using GenomeStudio Software (Illumina Inc.). Samples were grouped as follows: hES, iPS (iPS#12,iPS#3,iPS#4), EDK (EDK6,EDK7,EDK9), iPDK (iPDK2,iPDK3,iPDK4), BJ, NHK. Probe sets for each methylation site are specific for both methylated and unmethylated DNA fragments. Each methylation data point is represented as a β-value, corresponding to the fraction of cells within the total population with CpG methylation.

## Supporting Information

Figure S1 **Morphology of ES and iPS cells during multiple time-points of differentiation show similar morphology.** hES and hiPS cells were induced to differentiate in parallel, and monitored for cell morphology at various stages of differentiation in addition to those described in Figure 1 (A). iPS differentiation was morphologically analogous to ES differentiation in many areas of the differentiating culture, and the resultant cell populations were all positive for K18 (green). All images taken at 10X magnification. In addition to morphology, immunohistochemistry demonstrated the progressive loss of pluripotencyrelated surface markers TRA-1-60 and SSEA-4, and these markers was undetectable in differentiated cell types (B). Images taken at 20X magnification.
(TIF)

Figure S2 **Growth of EDK, iPDK, and BJ fibroblasts upon serial passage in culture.** The growth of EDK and iPDK cells was tracked over 12 passages and compared to normal fibroblasts. At each passage, cells were trypsinized, counted using a standard hemacytometer, and repassaged onto Type I collagen coated plates.
(TIF)
