# Supplementary figures and images for: Epigenetic and Phenotypic Profile of Fibroblasts Derived from Induced Pluripotent Stem Cells
Source: PLoS One. 2011 Feb 28;6(2):e17128. doi: 10.1371/journal.pone.0017128 (PMC3046119; doi:10.1371/journal.pone.0017128)

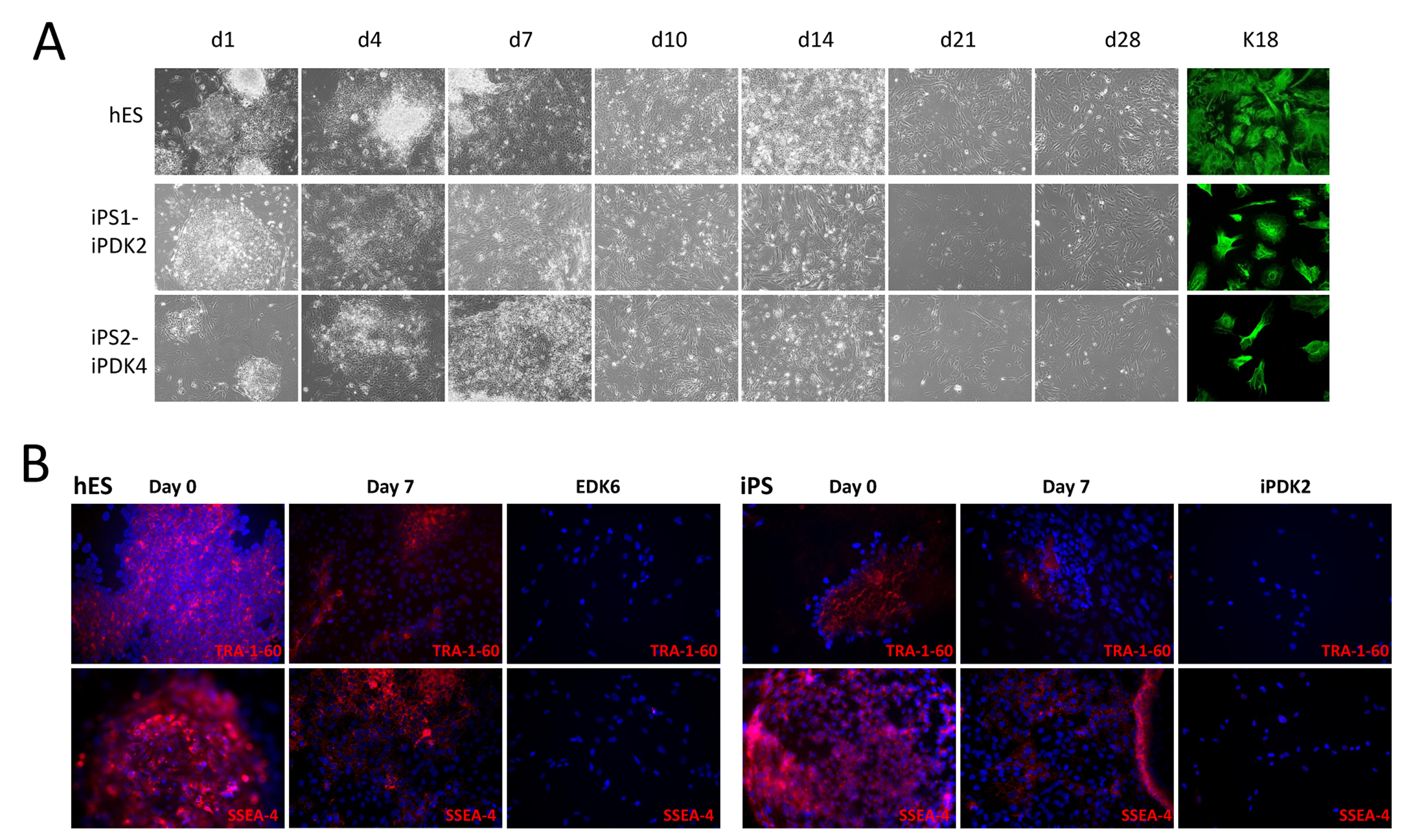

Supplement: Figure S1 — Morphology of ES and iPS cells during multiple time-points of differentiation show similar morphology. hES and hiPS cells were induced to differentiate in parallel, and monitored for cell morphology at various stages of differentiation in addition to those described in Figure 1 (A). iPS differentiation was morphologically analogous to ES differentiation in many areas of the differentiating culture, and the resultant cell populations were all positive for K18 (green). All images taken at 10X magnification. In addition to morphology, immunohistochemistry demonstrated the progressive loss of pluripotencyrelated surface markers TRA-1-60 and SSEA-4, and these markers was undetectable in differentiated cell types (B). Images taken at 20X magnification. (TIF) [file pone.0017128.s001.tif]

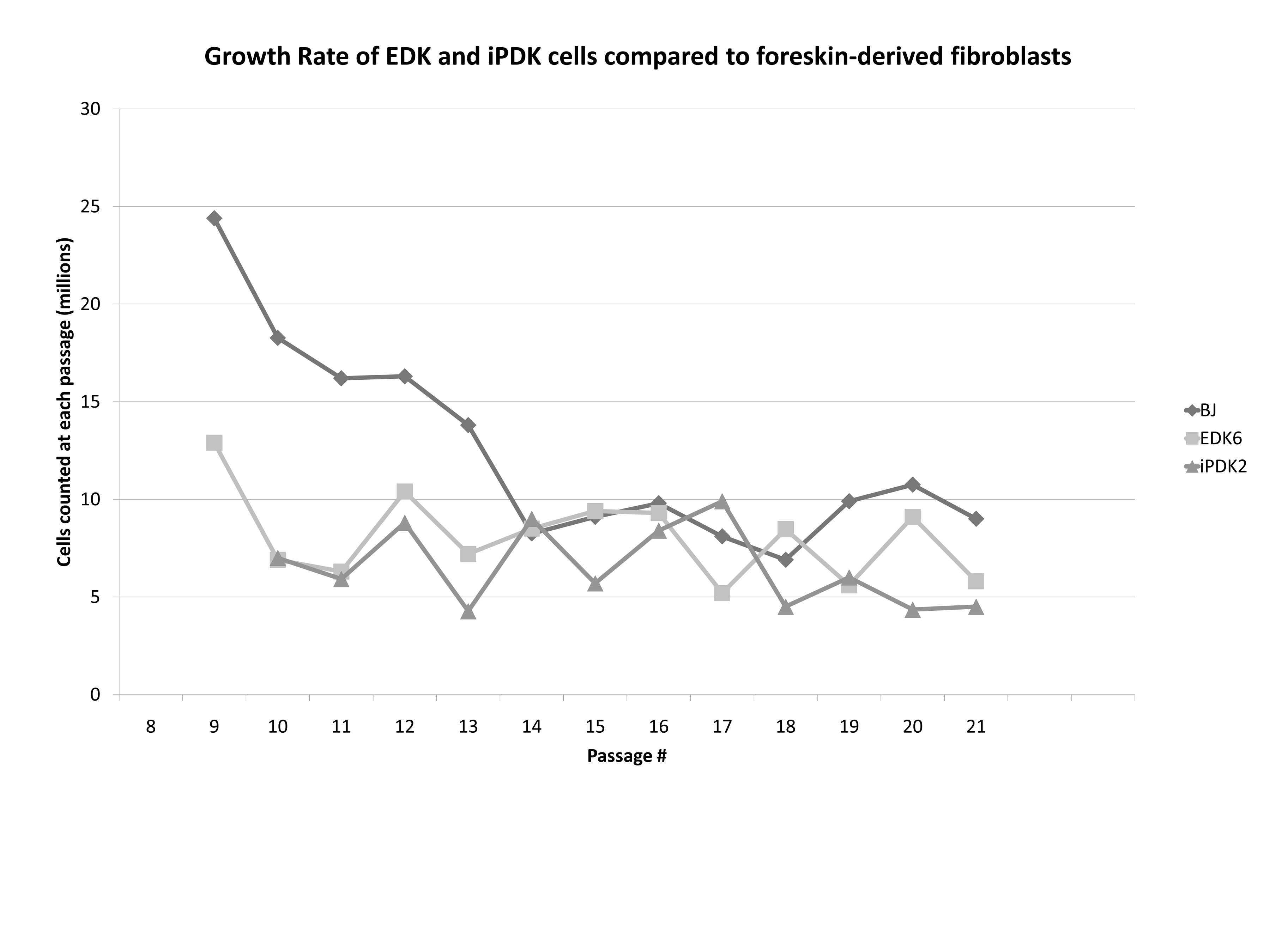

Supplement: Figure S2 — Growth of EDK, iPDK, and BJ fibroblasts upon serial passage in culture. The growth of EDK and iPDK cells was tracked over 12 passages and compared to normal fibroblasts. At each passage, cells were trypsinized, counted using a standard hemacytometer, and repassaged onto Type I collagen coated plates. (TIF) [file pone.0017128.s002.tif]
